# 3-(4-Nitro­phen­yl)-1*H*-1,2,4-triazole-5(4*H*)-thione

**DOI:** 10.1107/S1600536811033952

**Published:** 2011-08-27

**Authors:** Hoong-Kun Fun, Ching Kheng Quah, Nithinchandra, Balakrishna Kalluraya

**Affiliations:** a X-ray Crystallography Unit, School of Physics, Universiti Sains Malaysia, 11800 USM, Penang, Malaysia; b Department of Studies in Chemistry, Mangalore University, Mangalagangotri, Mangalore 574 199, India

## Abstract

In the title compound, C_8_H_6_N_4_O_2_S, the 1,2,4-triazole ring and the nitro group form dihedral angles of 6.26 (13) and 9.5 (3)°, respectively, with the phenyl ring. In the crystal, the mol­ecules are linked *via* pairs of N—H⋯S hydrogen bonds, generating [010] chains which contain *R* ^2^_2_ (8) ring motifs. The crystal structure is further stabilized by π–π stacking [centroid–centroid distance = 3.5491 (14) Å] inter­actions.

## Related literature

For general background to and the biological activity of 1,2,4-triazole derivatives, see: Shujuan *et al.* (2004 ▶); Clemons *et al.* (2004 ▶); Johnston (2002 ▶); Wei *et al.* (2007 ▶). For standard bond-length data, see: Allen *et al.* (1987 ▶). For the stability of the temperature controller used for the data collection, see: Cosier & Glazer (1986 ▶). For hydrogen-bond motifs, see: Bernstein *et al.* (1995 ▶). For related structures, see: Fun *et al.* (2010 ▶, 2011 ▶).
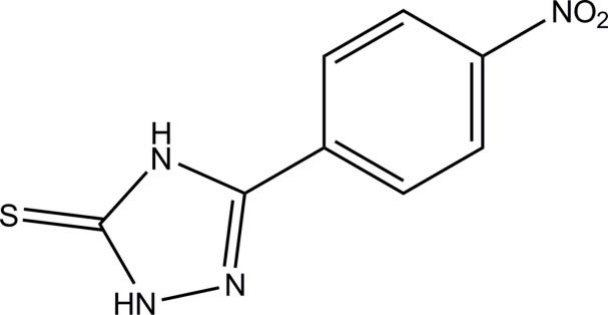


## Experimental

### 

#### Crystal data

C_8_H_6_N_4_O_2_S
*M* *_r_* = 222.23
Monoclinic, 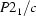

*a* = 7.8221 (1) Å
*b* = 8.2109 (1) Å
*c* = 14.6757 (3) Å
β = 101.302 (1)°
*V* = 924.29 (2) Å^3^
*Z* = 4
Mo *K*α radiation
μ = 0.33 mm^−1^
*T* = 100 K
0.35 × 0.27 × 0.17 mm

#### Data collection

Bruker SMART APEXII CCD diffractometer
Absorption correction: multi-scan (*SADABS*; Bruker, 2009 ▶) *T*_min_ = 0.892, *T*_max_ = 0.947
8521 measured reflections
1988 independent reflections
1789 reflections with *I* > 2σ(*I*)
*R*_int_ = 0.021

#### Refinement

*R*[*F* ^2^ > 2σ(*F* ^2^)] = 0.042
*wR*(*F* ^2^) = 0.109
*S* = 1.17
1988 reflections
144 parameters
H atoms treated by a mixture of independent and constrained refinement
Δρ_max_ = 0.42 e Å^−3^
Δρ_min_ = −0.29 e Å^−3^

### 

Data collection: *APEX2* (Bruker, 2009 ▶); cell refinement: *SAINT* (Bruker, 2009 ▶); data reduction: *SAINT*; program(s) used to solve structure: *SHELXTL* (Sheldrick, 2008 ▶); program(s) used to refine structure: *SHELXTL*; molecular graphics: *SHELXTL*; software used to prepare material for publication: *SHELXTL* and *PLATON* (Spek, 2009 ▶).

## Supplementary Material

Crystal structure: contains datablock(s) global, I. DOI: 10.1107/S1600536811033952/hb6376sup1.cif

Structure factors: contains datablock(s) I. DOI: 10.1107/S1600536811033952/hb6376Isup2.hkl

Supplementary material file. DOI: 10.1107/S1600536811033952/hb6376Isup3.cml

Additional supplementary materials:  crystallographic information; 3D view; checkCIF report

## Figures and Tables

**Table 1:** Hydrogen-bond geometry (Å, °)

*D*—H⋯*A*	*D*—H	H⋯*A*	*D*⋯*A*	*D*—H⋯*A*
N1—H1*N*1⋯S1^i^	0.84 (3)	2.48 (3)	3.295 (3)	164 (3)
N2—H1*N*2⋯S1^ii^	0.80 (3)	2.50 (3)	3.285 (3)	168 (3)

Symmetry codes: (i) 
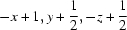
; (ii) 
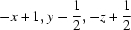
.

**Fig. 1.**
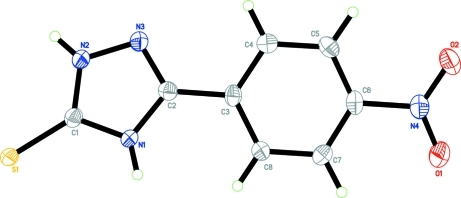
The molecular structure of the title compound showing 50% probability displacement ellipsoids for non-H atoms.

**Fig. 2.**
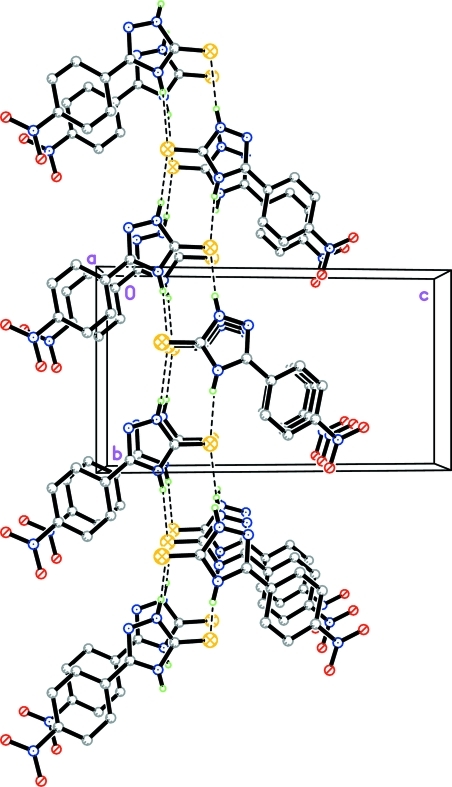
The crystal structure of the title compound, viewed along the a axis. H atoms not involved in hydrogen bonds (dashed lines) have been omitted for clarity.

## supplementary crystallographic information

### Comment

The 1,2,4-triazole nucleus has been incorporated into a wide variety of therapeutically interesting compounds. Several compounds containing 1,2,4-triazole rings are well known as drugs. For example, fluconazole is used as an antimicrobial drug (Shujuan *et al.*, 2004), whereas vorozole, letrozole and anastrozole are non-steroidal drugs used for the treatment of cancer (Clemons *et al.*, 2004) and loreclezole is used as an anticonvulsant (Johnston, 2002). Similarly substituted derivatives of triazole possess comprehensive bioactivities such as antimicrobial, anti-inflammatory, analgesic, antihypertensive, anticonvulsant and antiviral activities (Wei *et al.*, 2007). Due to the progress that occurs in dealing with the chemistry of 1,2,4-triazoles as well as their biological activity, we synthesized and reported the crystal structure of the title compound.

In the title molecule, Fig. 1, the 1,2,4-triazole ring (N1-N3/C1/C2, maximum deviation of 0.002 (2) Å at atoms N3 and C2) and the nitro group (O1/O2/N4) form dihedral angles of 6.26 (13) and 9.5 (3)°, respectively, with the phenyl ring (C3-C8). Bond lengths (Allen *et al.*, 1987) and angles are within normal ranges and are comparable to related structures (Fun *et al.*, 2010, 2011).

In the crystal structure, the molecules are linked *via* intermolecular N1–H1N1···S1 and N2–H1N2···S1 hydrogen bonds (Table 1), generating R^2^_2_ (8) ring motifs (Bernstein *et al.*, 1995) and are further linked into one-dimensional chains along [010] *via* adjacent ring motifs. π-π stacking interactions between the centroids of C3-C8 phenyl ring (Cg1) and N1-N3/C1/C2 triazole ring (Cg2), with Cg1···Cg2^iii^ distance of 3.5491 (14) Å [symmetry code: (iii) 1-X,1-Y,1-Z] are observed.

### Experimental

A mixture of 2-[(4-nitrophenyl)carbonyl]hydrazinecarbothioamide (0.01 mol) and 10% KOH (10 ml) was refluxed for 3 h. After the mixture was cooled to room temperature, it was then neutralized by the gradual addition of glacial acetic acid. The solid product obtained was collected by filtration, washed with ethanol and dried. It was then recrystallized using ethanol.  Yellow blocks of (I) were obtained from ethanol solution by slow evaporation.

### Refinement

Atoms H1N1 and H1N2 were located from the difference Fourier map and refined freely [N–H = 0.80 (3) or 0.84 (3) Å]. The remaining H atoms were positioned geometrically and refined using a riding model with C–H = 0.95 Å and *U*_iso_(H) = 1.2 *U*_eq_(C).

### Crystal data

**Table:**

C_8_H_6_N_4_O_2_S	*F*(000) = 456
*M**_r_* = 222.23	*D*_x_ = 1.597 Mg m^−^^3^
Monoclinic, *P*2_1_/*c*	Mo *K*α radiation, λ = 0.71073 Å
Hall symbol: -P 2ybc	Cell parameters from 6061 reflections
*a* = 7.8221 (1) Å	θ = 2.7–32.7°
*b* = 8.2109 (1) Å	µ = 0.33 mm^−^^1^
*c* = 14.6757 (3) Å	*T* = 100 K
β = 101.302 (1)°	Block, yellow
*V* = 924.29 (2) Å^3^	0.35 × 0.27 × 0.17 mm
*Z* = 4	

### Data collection

**Table:**

Bruker SMART APEXII CCD diffractometer	1988 independent reflections
Radiation source: fine-focus sealed tube	1789 reflections with *I* > 2σ(*I*)
graphite	*R*_int_ = 0.021
φ and ω scans	θ_max_ = 27.0°, θ_min_ = 2.7°
Absorption correction: multi-scan (*SADABS*; Bruker, 2009)	*h* = −9→8
*T*_min_ = 0.892, *T*_max_ = 0.947	*k* = −10→10
8521 measured reflections	*l* = −15→18

### Refinement

**Table:**

Refinement on *F*^2^	Primary atom site location: structure-invariant direct methods
Least-squares matrix: full	Secondary atom site location: difference Fourier map
*R*[*F*^2^ > 2σ(*F*^2^)] = 0.042	Hydrogen site location: inferred from neighbouring sites
*wR*(*F*^2^) = 0.109	H atoms treated by a mixture of independent and constrained refinement
*S* = 1.17	*w* = 1/[σ^2^(*F*_o_^2^) + (0.023*P*)^2^ + 1.7764*P*] where *P* = (*F*_o_^2^ + 2*F*_c_^2^)/3
1988 reflections	(Δ/σ)_max_ = 0.001
144 parameters	Δρ_max_ = 0.42 e Å^−^^3^
0 restraints	Δρ_min_ = −0.29 e Å^−^^3^

### Special details

**Table:**

Experimental. The crystal was placed in the cold stream of an Oxford Cryosystems Cobra open-flow nitrogen cryostat (Cosier & Glazer, 1986) operating at 100.0 (1) K.
Geometry. All esds (except the esd in the dihedral angle between two l.s. planes) are estimated using the full covariance matrix. The cell esds are taken into account individually in the estimation of esds in distances, angles and torsion angles; correlations between esds in cell parameters are only used when they are defined by crystal symmetry. An approximate (isotropic) treatment of cell esds is used for estimating esds involving l.s. planes.
Refinement. Refinement of F^2^ against ALL reflections. The weighted R-factor wR and goodness of fit S are based on F^2^, conventional R-factors R are based on F, with F set to zero for negative F^2^. The threshold expression of F^2^ > 2sigma(F^2^) is used only for calculating R-factors(gt) etc. and is not relevant to the choice of reflections for refinement. R-factors based on F^2^ are statistically about twice as large as those based on F, and R- factors based on ALL data will be even larger.

### Fractional atomic coordinates and isotropic or equivalent isotropic displacement parameters (Å^2^)

**Table:**

	*x*	*y*	*z*	*U*_iso_*/*U*_eq_	
S1	0.48563 (8)	0.37379 (7)	0.18859 (4)	0.02226 (18)	
O1	0.0046 (3)	0.9832 (3)	0.65011 (15)	0.0430 (6)	
O2	0.0293 (3)	0.7834 (3)	0.74650 (13)	0.0364 (5)	
N1	0.3673 (3)	0.5040 (3)	0.33704 (14)	0.0184 (4)	
N2	0.3841 (3)	0.2469 (3)	0.34126 (15)	0.0227 (5)	
N3	0.3212 (3)	0.2888 (3)	0.41922 (14)	0.0227 (5)	
N4	0.0471 (3)	0.8436 (3)	0.67295 (14)	0.0265 (5)	
C1	0.4131 (3)	0.3742 (3)	0.29010 (16)	0.0192 (5)	
C2	0.3128 (3)	0.4479 (3)	0.41483 (16)	0.0187 (5)	
C3	0.2506 (3)	0.5522 (3)	0.48290 (16)	0.0186 (5)	
C4	0.2100 (3)	0.4814 (3)	0.56284 (16)	0.0206 (5)	
H4A	0.2269	0.3680	0.5738	0.025*	
C5	0.1450 (3)	0.5774 (3)	0.62606 (16)	0.0218 (5)	
H5A	0.1165	0.5310	0.6805	0.026*	
C6	0.1226 (3)	0.7419 (3)	0.60807 (16)	0.0223 (5)	
C7	0.1638 (3)	0.8162 (3)	0.53043 (17)	0.0235 (5)	
H7A	0.1490	0.9301	0.5208	0.028*	
C8	0.2272 (3)	0.7193 (3)	0.46708 (16)	0.0210 (5)	
H8A	0.2548	0.7668	0.4127	0.025*	
H1N1	0.384 (4)	0.601 (4)	0.323 (2)	0.026 (8)*	
H1N2	0.400 (4)	0.154 (4)	0.329 (2)	0.033 (9)*	

### Atomic displacement parameters (Å^2^)

**Table:**

	*U*^11^	*U*^22^	*U*^33^	*U*^12^	*U*^13^	*U*^23^
S1	0.0335 (3)	0.0136 (3)	0.0225 (3)	−0.0009 (2)	0.0124 (2)	−0.0021 (2)
O1	0.0675 (15)	0.0288 (12)	0.0374 (11)	0.0141 (11)	0.0220 (10)	−0.0025 (9)
O2	0.0480 (12)	0.0404 (13)	0.0235 (9)	0.0060 (10)	0.0140 (8)	−0.0005 (9)
N1	0.0223 (10)	0.0137 (11)	0.0204 (10)	0.0009 (8)	0.0070 (8)	−0.0006 (8)
N2	0.0280 (11)	0.0160 (11)	0.0271 (11)	0.0011 (9)	0.0128 (9)	−0.0006 (9)
N3	0.0262 (10)	0.0192 (11)	0.0255 (10)	0.0011 (8)	0.0118 (8)	−0.0001 (9)
N4	0.0292 (11)	0.0280 (13)	0.0229 (10)	0.0014 (9)	0.0063 (8)	−0.0047 (9)
C1	0.0185 (10)	0.0170 (12)	0.0222 (11)	−0.0003 (9)	0.0045 (9)	−0.0029 (10)
C2	0.0149 (10)	0.0207 (13)	0.0207 (11)	0.0002 (9)	0.0040 (8)	0.0016 (10)
C3	0.0146 (10)	0.0217 (13)	0.0198 (11)	0.0000 (9)	0.0040 (8)	−0.0044 (10)
C4	0.0188 (10)	0.0205 (13)	0.0220 (11)	−0.0007 (9)	0.0026 (9)	0.0014 (10)
C5	0.0193 (11)	0.0287 (14)	0.0170 (11)	−0.0006 (10)	0.0030 (9)	0.0014 (10)
C6	0.0197 (11)	0.0272 (14)	0.0202 (11)	0.0015 (10)	0.0046 (9)	−0.0060 (10)
C7	0.0264 (12)	0.0187 (12)	0.0258 (12)	0.0037 (10)	0.0065 (10)	−0.0002 (10)
C8	0.0241 (11)	0.0199 (13)	0.0205 (11)	−0.0005 (9)	0.0079 (9)	−0.0003 (10)

### Geometric parameters (Å, °)

**Table:**

S1—C1	1.695 (2)	C2—C3	1.469 (3)
O1—N4	1.222 (3)	C3—C8	1.398 (3)
O2—N4	1.220 (3)	C3—C4	1.400 (3)
N1—C1	1.355 (3)	C4—C5	1.387 (3)
N1—C2	1.375 (3)	C4—H4A	0.9500
N1—H1N1	0.84 (3)	C5—C6	1.380 (4)
N2—C1	1.332 (3)	C5—H5A	0.9500
N2—N3	1.375 (3)	C6—C7	1.385 (3)
N2—H1N2	0.80 (3)	C7—C8	1.387 (3)
N3—C2	1.309 (3)	C7—H7A	0.9500
N4—C6	1.474 (3)	C8—H8A	0.9500
			
C1—N1—C2	108.3 (2)	C8—C3—C2	120.6 (2)
C1—N1—H1N1	124 (2)	C4—C3—C2	119.2 (2)
C2—N1—H1N1	127 (2)	C5—C4—C3	119.8 (2)
C1—N2—N3	113.7 (2)	C5—C4—H4A	120.1
C1—N2—H1N2	125 (2)	C3—C4—H4A	120.1
N3—N2—H1N2	122 (2)	C6—C5—C4	118.5 (2)
C2—N3—N2	103.4 (2)	C6—C5—H5A	120.8
O2—N4—O1	123.4 (2)	C4—C5—H5A	120.8
O2—N4—C6	118.1 (2)	C5—C6—C7	123.2 (2)
O1—N4—C6	118.4 (2)	C5—C6—N4	118.8 (2)
N2—C1—N1	103.9 (2)	C7—C6—N4	118.0 (2)
N2—C1—S1	128.14 (19)	C6—C7—C8	118.1 (2)
N1—C1—S1	127.98 (19)	C6—C7—H7A	121.0
N3—C2—N1	110.8 (2)	C8—C7—H7A	121.0
N3—C2—C3	124.6 (2)	C7—C8—C3	120.3 (2)
N1—C2—C3	124.6 (2)	C7—C8—H8A	119.9
C8—C3—C4	120.2 (2)	C3—C8—H8A	119.9
			
C1—N2—N3—C2	−0.3 (3)	C2—C3—C4—C5	−177.6 (2)
N3—N2—C1—N1	0.1 (3)	C3—C4—C5—C6	−0.2 (3)
N3—N2—C1—S1	−178.54 (17)	C4—C5—C6—C7	−0.7 (4)
C2—N1—C1—N2	0.1 (2)	C4—C5—C6—N4	177.6 (2)
C2—N1—C1—S1	178.73 (18)	O2—N4—C6—C5	9.9 (3)
N2—N3—C2—N1	0.3 (3)	O1—N4—C6—C5	−169.7 (2)
N2—N3—C2—C3	179.3 (2)	O2—N4—C6—C7	−171.7 (2)
C1—N1—C2—N3	−0.2 (3)	O1—N4—C6—C7	8.7 (3)
C1—N1—C2—C3	−179.2 (2)	C5—C6—C7—C8	1.3 (4)
N3—C2—C3—C8	−172.6 (2)	N4—C6—C7—C8	−177.0 (2)
N1—C2—C3—C8	6.3 (3)	C6—C7—C8—C3	−0.9 (4)
N3—C2—C3—C4	5.6 (4)	C4—C3—C8—C7	0.0 (3)
N1—C2—C3—C4	−175.6 (2)	C2—C3—C8—C7	178.2 (2)
C8—C3—C4—C5	0.5 (3)		

### Hydrogen-bond geometry (Å, °)

**Table:**

*D*—H···*A*	*D*—H	H···*A*	*D*···*A*	*D*—H···*A*
N1—H1N1···S1^i^	0.84 (3)	2.48 (3)	3.295 (3)	164 (3)
N2—H1N2···S1^ii^	0.80 (3)	2.50 (3)	3.285 (3)	168 (3)

Symmetry codes: (i) −*x*+1, *y*+1/2, −*z*+1/2; (ii) −*x*+1, *y*−1/2, −*z*+1/2.

## References

[bb1] Allen, F. H., Kennard, O., Watson, D. G., Brammer, L., Orpen, A. G. & Taylor, R. (1987). *J. Chem. Soc. Perkin Trans. 2*, pp. S1–S19.

[bb2] Bernstein, J., Davis, R. E., Shimoni, L. & Chang, N.-L. (1995). *Angew. Chem. Int. Ed. Engl.* **34**, 1555–1573.

[bb3] Bruker (2009). *APEX2*, *SAINT* and *SADABS* Bruker AXS Inc., Madison, Wisconsin, USA.

[bb4] Clemons, M., Coleman, R. E. & Verma, S. (2004). *Cancer Treat. Rev.* **30**, 325–332. 10.1016/j.ctrv.2004.03.004 15145507

[bb5] Cosier, J. & Glazer, A. M. (1986). *J. Appl. Cryst.* **19**, 105–107.

[bb6] Fun, H.-K., Quah, C. K., Nithinchandra & Kalluraya B. (2011). *Acta Cryst.* E**67**, o2416. 10.1107/S1600536811033940 PMC3200591 22059001

[bb7] Fun, H.-K., Quah, C. K., Vijesh, A. M., Malladi, S. & Isloor, A. M. (2010). *Acta Cryst.* E**66**, o29–o30. 10.1107/S1600536809051368 PMC2980099 21580136

[bb8] Johnston, G. A. R. (2002). *Curr. Top. Med. Chem.* **2**, 903–913. 10.2174/1568026023393453 12171579

[bb9] Sheldrick, G. M. (2008). *Acta Cryst.* A**64**, 112–122. 10.1107/S0108767307043930 18156677

[bb10] Shujuan, S., Hongxiang, L., Gao, Y., Fan, P., Ma, B., Ge, W. & Wang, X. (2004). *J. Pharm. Biomed. Anal.* **34**, 1117–1124. 10.1016/j.jpba.2003.11.013 15019046

[bb11] Spek, A. L. (2009). *Acta Cryst.* D**65**, 148–155. 10.1107/S090744490804362X PMC2631630 19171970

[bb12] Wei, T.-B., Tang, J., Liu, H. & Zhang, Y.-M. (2007). *Phosphorus Sulfur Silicon*, **182**, 1581–1587.

